# Clinical presentations and outcomes of Filipino juvenile systemic lupus erythematosus

**DOI:** 10.1186/1546-0096-9-7

**Published:** 2011-02-09

**Authors:** Carien B Gulay, Leonila F Dans

**Affiliations:** 1 Section of Pediatric Rheumatology, Department of Pediatrics, Philippine General Hospital-University of the Philippines Manila, Taft Avenue, Manila, Philippines

## Abstract

**Objective:**

Juvenile Systemic Lupus Erythematosus (SLE) varies by location and ethnicity. This study describes the clinical, laboratory profile and outcome of juvenile SLE seen at Philippine General Hospital (PGH) from 2004-2008.

**Method:**

Medical charts of all Filipino Juvenile SLE cases admitted at PGH during the 5-year period were reviewed collecting demographic profile, clinical and laboratory manifestations and treatment during disease course.

**Results:**

Seventy-eight cases of juvenile SLE were reviewed. There were 7 boys and 71 girls. The mean age at diagnosis was 14 years (SD 2.7) with a range of 8-18 years. Fever (52.5%) and malar rash (41.0%) were the most common features at disease onset. At the time of diagnosis, the most common features were malar rash (65.3%), renal involvement (62.8%) and photosensitivity (55.1%). Mucocutaneous (92.3%), renal (71.7%) and hematologic (69.2%) involvement were the most common features during the entire course of illness. Infection (34.5%) and neurologic (19.0%) complications were observed most frequently. Corticocosteroid treatment was given in most of the patients in the form of prednisone (97.4%) and concomitant methylprednisolone intravenous pulses (29.4%). Nine patients died during the study period. The overall 5-year mortality rate was 11.5%. Infection (77.0%) was the most frequent cause of death.

**Conclusion:**

Malar rash was a common feature at disease onset and at diagnosis among Filipinos with juvenile SLE. Throughout the disease course, renal involvement occurs in 71.7% of patients. Infection was the leading cause of complication and death. The clinical presentations of Filipinos with juvenile SLE were similar to juvenile SLE in other countries.

## Introduction

Systemic Lupus Erythematosus (SLE) is an autoimmune disease characterized by widespread inflammation of blood vessels and connective tissues and by the presence of antinuclear antibodies (ANAs), especially antibodies to native double stranded DNA (Anti dsDNA). Its clinical manifestation is variable and its natural history is unpredictable. Incidence of juvenile SLE varies by location and ethnicity. There is a wide range of variation in the natural history of SLE among different ethnic and geographical groups. Studies in juvenile SLE have estimated the incidence at 0.28 to 0.9/100,000 per year [1-3]. A previous study done by Brunner et al in 2008 among Caucasians suggests that patients with childhood-onset SLE have more active disease upon presentation and over time than adults. Children received more intensive drug therapy and accrue more damage [4]. Two significant factors with poor prognosis are female gender and development of end-stage renal disease (ESRD) [5]. Organ damage was significantly more likely to occur in patients who experienced neuropsychiatric manifestations upon diagnosis, had longer disease duration, and had received more intravenous pulses of cyclophosphamide [6]. Recurrent major infections significantly correlate with poorer long-term organ damage [7].

The actual prevalence of juvenile SLE among Filipinos is still unknown. With the review of the clinical manifestations of SLE cases and increased awareness of this condition in pediatric patients, this study hopes to be of value in ensuring early recognition and early institution of appropriate management of this often missed and mismanaged condition. We chose to review cases at the Philippine General Hospital (PGH), a tertiary government hospital located in Manila Philippines. It is the Filipino people's national university hospital and premier referral center. It caters more for low-income patients and it is the only institution in the country with a Pediatric Rheumatology training program.

This retrospective study aims to describe the clinical, laboratory profile and outcome of juvenile SLE seen at the PGH during a 5-year period from January 2004 to December 2008.

### Methodology

Patients diagnosed with juvenile SLE (met > 4 criteria of the 1997 revised American College of Rheumatology [ACR] classification criteria for SLE); age at onset <18 years according to the Paediatric Rheumatology International Trials Organization (PRINTO) [8] were included in the study. Patients were identified from the Out-patient and In-patient (wards and emergency room) service of General Pediatrics, Pediatric Rheumatology and Pediatric Nephrology sections of PGH. The institutional review board approved the study.

Medical charts were retrieved from the records section of PGH by the primary investigator (CG). These were reviewed from the date of the initial consult up to the last date of follow up. A data collection form was used to collect information on demographic profile, presenting features, clinical and laboratory manifestations and treatment during the course of the disease for each patient.

Clinical presentation at onset of disease was defined as the manifestations at the first 3 months of illness. The time of diagnosis was defined as the time when the patient fulfilled at least four ACR 1997 revised classification criteria for SLE.

Records of clinical and laboratory findings during the study period were collected as follows: Hemolytic anemia with reticulocytosis, leucocytopenia (< 4000 cells/mm3) and lymphopenia [absolute lymphocytic count (ALC) <1,500 cells/mm^3 ^] present in 2 or more occasions, thrombocytopenia (< 100,000 cells/mm^3^) in the absence of offending drugs, abnormal urinalysis (proteinuria 0.5 g/24 h or >3+ if quantitative evaluation was not done, and/or casts, hematuria >5 RBCs/HPF or pyuria >5 WBCs/HPF in the absence of infection), increased creatinine (> 1 mg/dl), low C3, increased erythrocyte sedimentation rate (ESR) (> 20 mm/hr). Pleuritis documented by history of pleuritic pain or rub heard by physician or evidence of pleural effusion and pericarditis documented by ECG, rub, or evidence of pericardial effusion. Growth failure is defined as the presence of at least 2 of the following 3 features: 1) height below the third percentile for age; 2) growth velocity over 6 months below the third percentile for age; 3) crossing at least 2 percentiles (5%,10%, 25%, 50%, 75%, 95%) on National Center for Health Statistics growth charts. Delayed puberty is defined as a delay in development of secondary sexual characteristics more than 2 SD below the mean for age by Tanner staging. Complications and causes of death were analyzed. Descriptive statistics which include mean, median, range and frequency were computed.

## Results

Of the 84 children diagnosed with juvenile SLE seen in our institution, 78 of them (93.0%) had available medical records for review. The female to male ratio was 10:1 (71 girls, 7 boys). The overall mean age at diagnosis was 14.0 years (SD 2.7) (boys 13.7 years, girls 14.0 years), with a range of 8-18 years. The peak age group on diagnosis is 11 year old for male and 17 year old for female patients. Eight (10.2%) of our Filipino juvenile SLE patients are known to have history of SLE in the family. Six of them were first-degree relatives.

### Clinical and Laboratory Features

Fever (52.5%), malar rash (41.0%), oral/nasopharyngeal ulcers (29.4%), alopecia and general fatigue (28.2%) were the most common features at disease onset (see Table 1).

**Table 1:** Presenting features at Disease Onset among 78 juvenile SLE patients

Manifestation	**No**.	%
Mucocutaneous		

Malar rash	32	41.0

Oral/nasopharyngeal ulcers	23	29.4

Alopecia	22	28.2

Photosensitivity	19	24.0

Punctate erythema	7	8.9

Discoid rash	5	6.4

Epistaxis	5	6.4

Gumbleeding	2	2.5

Musculoskeletal		

Arthralgia	21	26.9

Arthritis	14	17.9

Haematologic		

Pallor	16	20.5

Neuropsychiatric		

Seizures	3	3.8

Psychiatric symptoms	2	2.5

Renal		

Gross hematuria	9	11.5

Gastrointestinal		

Abdominal pain	7	8.9

Vomiting	7	8.9

Constitutional		

Fever	41	52.5

General fatigue	22	28.2

Weight loss	14	17.9

Loss of appetite	7	8.9

At the time of diagnosis, the most common features were malar rash (65.3%), renal involvement (62.8%) and photosensitivity (55.1%) (see Table 2). Cardiovascular involvement was found in 17.0% of patients, pericardial effusion was noted in 15.3% and valvular anomaly 10.2%. They presented with tricuspid regurgitation (5), pulmonary regurgitation (2), mitral regurgitation (2) and moderate aortic regurgitation (1). Hematologic manifestations were as follows: 58.0% had anemia (hemoglobin range: 3.6-11.8; median: 9.35; mean 8.86 SD 2.02 g/dl), 8.9% with positive Coombs test, 15.3% with leukocytopenia (range: 2,950-3,900; median 3,800 cell/mm^3^), 21.7% with lymphocytopenia (ALC range: 325-1,431; median 1,034 cells/mm^3^), 14.1% with thrombocytopenia (platelet count range: 27,000-90,000; median 56,000 cells/mm^3^).

**Table 2:** Clinical and Laboratory Features among 78 juvenile SLE patients

Category	At the time of diagnosis		during the disease course	
	
	**No**.	%	**No**.	%
**Mucocutaneous** **Manifestations**	**71**	**91.0**	**72**	**92.3**

Malar rash	51	65.3	60	76.9

Discoid rash	25	32.0	28	35.8

Photosensitivity	43	55.1	57	73.0

Oral ulcers	42	53.8	53	67.9

Alopecia	31	39.7	41	52.5

**Musculoskeletal** **Involvement**	**32**	**41.0**	**42**	**53.8**

Arthritis	17	21.7	31	39.7

Arthralgia	24	30.7	40	51.2

**Pulmonary Involvement**	**11**	**14.1**	**17**	**21.7**

Pleuritis/Pleural effusion	11	14.1	16	20.5

**Cardiac Involvement**	**13**	**16.6**	**17**	**21.7**

Pericarditis/Pericardial Effusion	12	15.3	14	20.5

Valvular anomaly on Echocardiography	8	10.2	8	10.2

Ventricular/atrial Hypertrophy	4	5.1	5	6.4

**Renal involvement**	**49**	**62.8**	**56**	**71.7**

Hematuria	19	24.3	26	33.3

Proteinuria	32	41.0	20	25.6

**CNS involvement**	**24**	**30.7**	**25**	**32.0**

Seizure	12	15.3	15	19.2

Behavioral changes	9	11.5	10	12.8

**Hematologic** **Involvement**	**37**	**47.4**	**54**	**69.2**

**Laboratory**				

Cellular casts	11	14.1	22	28.2

Increased creatinine	6	7.6	11	14.1

Hemolytic anemia	7	8.9	8	10.2

Leukocytopenia	12	15.3	25	32.0

Lymphocytopenia	17	21.7	32	41.0

Thrombocytopenia	11	14.1	20	25.6

Low C3	7	8.9	7	8.9

Mucocutaneous (92.3%), renal (71.7%) and hematologic (69.2%) involvement were the most common features during the entire course of illness(see Table 2).

ANA was positive in 98.5% (n = 70/71) of our patients, mostly done using Indirect Immunofluorescence (IIF) technique using HEp-2 cells (70.0%). LE cell could be demonstrated in 25.6% of patients. Anti-dsDNA antibodies were detected by IIF on *Crithidiae luciliae*. It was done in only 21 patients and titers were significantly high among 18 of them.

The frequency of complications in our juvenile SLE patients is shown in Table 3. Infection (34.5%) followed by neurologic (19.0%) and musculoskeletal (8.7%) were observed most frequently.

**Table 3:** Complications among 78 juvenile SLE patients

Item	N	%
**Infection**	**27**	**34.5**
Pneumonia	11	14.1
Sepsis	8	10.2
Tuberculosis (pulmonary, endotracheal, miliary)	6	7.7
Cellulitis	2	2.5

**Neurologic**	**15**	**19.0**
Seizure requiring therapy for 6 months	12	15.3
Cerebral atrophy by imaging (Cranial CT scan)	2	2.5
Lateral rectus palsy	1	1.2

**Musculoskeletal**	**5**	**8.7**
Deforming or erosive arthritis	3	3.8
Osteoporosis with fracture or vertebral collapse	2	2.5
Osteomyelitis	1	1.2
Muscle atrophy or weakness	1	1.2

**Skin**	**4**	**5.0**
Scarring chronic alopecia	2	2.5
Extensive scarring or panniculum other than scalp and pulp space	2	2.5

**Gastrointestinal**	**2**	**2.0**
Autoimmune hepatitis by biopsy	2	2.0

**Renal**	**2**	**2.5**
Estimated or measured glomerular filtration rate < 50%	2	2.5

**Growth failure**	**4**	**5.1**

**Pubertal delay**	**2**	**2.5**

**Ocular**	**1**	**1.2**
Ischemic retinopathy	1	1.2

**Pulmonary**	**1**	**1.2**
Pulmonary hypertension	1	1.2

Throughout the study period, 71.7% (56/78) of patients had renal involvement. Renal biopsy was done only among 24 of these patients. Evaluation was based on WHO classification for lupus nephritis. One was found to be of mixed type and was then classified under the dominant class. Renal histology showed class II nephritis in 25.0%, class III in 20.8%, class IV in 50.0% and class V in 4.1%.

### Therapy

Corticosteroid treatment was given in most of the patients in the form of prednisone (97.4%) and Methylprednisolone IV pulses (29.4%). Hydroxychloroquine was used in 32.0%, Azathioprine in 23.0%, Cyclophosphamide intravenous pulses in 26.9%.

### Clinical Outcomes

The follow-up period ranged from 0.1-6.4 years with a mean duration of 1.7 years (SD 1.8). About 34.6% of the Filipino juvenile SLE patients seen our institution are still attending our clinics, 21.7% were endorsed to adult rheumatology clinic, 6.4% transferred to other institution while 25.6% were lost to follow-up.

Nine patients died during the five-year study period. The causes of death were (1) active lupus (acute renal failure), (1) severe pulmonary hypertension and (7) infection. Identified pathogens on blood culture were *Pseudomonas aeroginosa*, Methicillin-resistant *Staphylococcus epidermidis, Burkholderia mallei and Klebsiella pneumoniae*. One had concomitant staphylococcal pneumonia and the other with osteomyelitis secondary to *Salmonella sp *(documented by synovial fluid culture).

## Discussion

We compared the manifestations at diagnosis as well as the cumulative features of our juvenile SLE patients with the local data as well as from other countries (see Table 4 and 5). Available literatures with hospital-based data on juvenile SLE were included.

**Table 4:** Clinical And Laboratory Variables Of Children With SLE At Diagnosis Compared To Other Countries

	Present Study	Wang, 2003^5^	Balkaran, 2004^10^	Bakr, 2005^11^	Salah, 2009^9^	Hamijoyo, 2009^12^
**No.of** **Patients**	78	153	33	52	207	147

**Country**	Philippines	Taiwan	Trinidad	Egypt	Egypt	Philippines

**Follow-up, y**						

**Mean** **Range**	1.7 ± 1.8 [0.1-6.4]	6.1 ± 9.0		1 ± 9.3		

**Age at diagnosis, y** **Mean+SD** **Range**	14 ± 2.7 8 - 18	13.5 ± 5.5	5-17	11.9 ± 2.6 6-16	10 ± 2.7 2 - 16	12.29 ± 2.9 4 - 16

**F:M ratio**	10:1	5.9:1	6.6:1	12:1	2.69:1	11.2:1

**Rash**						

**Malar**	65.3	77.1	39	46.2		74.8

**Discoid**	32.0	2.0	37			19.0

**Photosensitivity**	55.1	24.8		21,2		57.8

**Oral ulcers**	53.8	26.1		19.6		49.7

**Alopecia**	39.7	13.1		34.6	45.5	51.7

**Musculoskeletal**	53.8		69.7	65.4		

**Arthritis**	21.7	57.5			46.9	61.9

**Neuropsychiatric**	30.7	4.6		7.7	7.2	8.2

**Serositis**	26.9	15				14.3

**Pleural** **Effusion**	14.1				6.3	

**Pericardial** **Effusion**	15.3			7.7		

**Nephritis**	62.8	58.8	63.6	80.8	20.8	48.3

**Hematologic**	51.2	79.7				38.8

**Thrombocytopenia**	14.1	19.6		29.2		

**Hemolytic** **Anemia**	8.9	44.4			17.0	

**Leukocytopenia**	15.3	34.6		27.5		

**Table 5:** Cumulative Features Of Children With SLE Compared To Other Countries

Cumulative Features	Present Study n = 78 (%)	Agcaoili 1986^13^ n = 31(%)	Bahabri, 1997 n = 60^14 ^(%)	Gedalia, 1999^15^ n = 61 (%)		Pattaragarn, 2005^16^ n = 82 (%)	Hiraki, 2008^17^ n = 256 (%)	Salah, 2009^9^ n = 207 (%)	Hoffman2009^18^ n = 56(%)
**Country**	Philippines	Philippines	Saudi Arabia	African-America	Latin America	Thailand	Canada	Egypt	Europe

**Rash**	75.6			84	81				

**Malar**	71.7	90.3	40	69	52	53.5	66	38.2	69.6

**Discoid**	35.8			21	9	2		10.1	13.2

**Photosensitivity**	73		15	20	56	21.8		44	44.6

**Oral ulcers**	67.9	45.1	16			31.7		22.2	28.6

**Musculoskeletal** **Involvement**	53.8		91.6			31.7		39.6	

**Arthritis**	39.7	74.1		79	75		67		59.3

**Neuropsychiatric**	32	32.2 psych 25.8 sz	27	31	40	20.8	27	24.2	

**Hematologic involvement**	69.2		66.6			73.4			

**Hemolytic** **anemia**	10.2			18	12			19.3	38.5

**Thromobocyto** **penia**	25.6	6.4		26	22	13.9		21.7	31.5

**Leukocytopenia**	32	12.9		46	55			26	63.6

**Serositis**	26.9								

**Carditis**	17.9	32.26		28	16				16.7

**Pleuritis**	20.5	25.81		36	24				

**Renal** **Involvement**	71.7		65	44	55	86.2	55	67	62.5

In our series, mean age at diagnosis was at 14.0 (SD 2.7) years old which is comparable to other studies (see Table 5). We reported 8 yr old as the youngest age at initial SLE diagnosis. This is relatively old compared to other series with reported youngest age at 2 to 7 years old [9-12]. Female preponderance, similar from reports of other countries, emphasizes the importance of hormonal factors in the clinical expression of the disease.

All local studies showed malar rash as the most common clinical feature at the time of diagnosis as well as during the entire course of illness [12,13]. Our patients had fewer reports of arthritis but a significantly higher neuropsychiatric manifestations compared to the other local study done by Hamijoyo[12,13] as well as with that of Taiwan [5] and Egypt [9]. The frequency of renal involvement in our study (62.8%) was comparable to the studies from Taiwan (58.8%) [5] and Trinidad (63.6%)[10]. Among the clinical features found throughout the entire clinical course, the cumulative frequency of hematologic (69.2%) and neuropsyschiatric (32%) involvement in our series is comparable to other studies [9,13-16]. However, arthritis still remained low throughout the course of illness in our patient population compared to other studies [13,14,16,17]. The cumulative frequency of renal involvement in our series (71.7%) is comparable to studies involving Arab (65%) [14] and Egyptian (67%) [5] children but more common than in Canadian (55.0%) [17], European (62.5%) [18], African-American (44.0%) [15] and Latin American children (55%) [15]. Wide variations among different studies may be attributed to genetic differences or to referral bias, as some studies came from nephrology units and others from rheumatology units.

Most of our patients with lupus nephritis had pathological changes consistent with Class III and IV lupus nephritis (WHO classification). Report of nephritis was highest in Thailand (86.2%)[16] and lowest in African-American children(44%)[15]. These diverse presentations, disease course and outcome appear to be multifactorial. Environmental, socioeconomic, demographic, psychosocial, genetic (HLA-DRBl*0301, HLA-DRB1, FCGR gene family, IRF5, STAT4 and MECP2) [19], and clinical factors play an important role as determinants of the ethnic differences [20]. Further investigation is needed to elucidate the basis of these disparities.

One of the limitations of this study was that not all patients were able to have the complete antibody profile and kidney biopsy. This was primarily due to financial reasons as majority of our patients came from low income families.

Of the 9 patients who died the most common cause was infection (77%). The identified pathogens included were mostly gram negative bacteria. Nosocomial infection was the predominant type. This was similar in most studies on juvenile SLE, as infection has replaced renal failure as the leading cause of mortality among these patients [5,21-23]. Use of immunosuppressive therapy and inherent immune abnormalities in active lupus predisposes these children to infection. However, a major contributory factor which probably caused such high mortality in our institution could be that most of our patients belong to the low income families contributing to poor treatment compliance and adherence and poor follow-up. Another potential factor could be that our patients might have more severe disease spectrum since we are the biggest government institution with a referral center for children with rheumatologic diseases. Faced with such obstacles, a stronger awareness and recognition of this condition by other health professionals hopefully would assure early referral to the pediatric rheumatologist and other appropriate subspecialists. Emphasis in programs for health promotions among chronically ill patients and access support group for Filipino children suffering from lupus should also be advocated.

## Conclusion

Malar rash was a common feature at disease onset and at diagnosis among Filipinos with juvenile SLE. Throughout the disease course, renal involvement occurs in 71.7% of patients. Infection was the leading cause of complication and death. The clinical presentations of Filipinos with juvenile SLE were similar to juvenile SLE in other countries.

## Competing interests

The authors declare that they have no competing interests.

## Authors' contributions

CG and LD - substantial contribution to conception and design. Each of them participated sufficiently in preparation of this Manuscript. All authors read and approved the final manuscript.
